# Sigma-1 Receptor Changes Observed in Chronic Pelvic Pain Patients: A Pilot PET/MRI Study

**DOI:** 10.3389/fpain.2021.711748

**Published:** 2021-10-20

**Authors:** Daehyun Yoon, Angela M. Fast, Peter Cipriano, Bin Shen, Jessa B. Castillo, Christopher R. McCurdy, Carina Mari Aparici, Deirdre Lum, Sandip Biswal

**Affiliations:** ^1^Department of Radiology, School of Medicine, Stanford University, Stanford, CA, United States; ^2^Diagnostic, Molecular and Interventional Radiology, The Mount Sinai Hospital, New York, NY, United States; ^3^Department of Medicinal Chemistry, College of Pharmacy, University of Florida, Gainesville, FL, United States; ^4^Department of Obstetrics and Gynecology, School of Medicine, Stanford University, Stanford, CA, United States

**Keywords:** chronic pelvic pain, sigma-1 receptor, positron emission tomography, magnetic resonance imaging, PET/MRI

## Abstract

**Introduction:** Chronic pelvic pain is a highly prevalent pain condition among women, but identifying the exact cause of pelvic pain remains a significant diagnostic challenge. In this study, we explored a new diagnostic approach with PET/MRI of the sigma-1 receptor, a chaperone protein modulating ion channels for activating nociceptive processes.

**Methods:** Our approach is implemented by a simultaneous PET/MRI scan with a novel radioligand [18F]FTC-146, which is highly specific to the sigma-1 receptor. We recruited 5 chronic pelvic pain patients and 5 healthy volunteers and compared our PET/MRI findings between these two groups.

**Results:** All five patients showed abnormally increased radioligand uptake on PET compared to healthy controls at various organs, including the uterus, vagina, pelvic bowel, gluteus maximus muscle, and liver. However, on MRI, only 2 patients showed abnormalities that could be potentially associated with the pain symptoms. For a subset of patients, the association of pain and the abnormally increased radioligand uptake was further validated by successful pain relief outcomes following surgery or trigger point injections to the identified abnormalities.

**Conclusion:** In this preliminary study, sigma-1 receptor PET/MRI demonstrated potential for identifying abnormalities associated with chronic pelvic pain. Future studies will need to correlate samples with imaging findings to further validate the correlation between S1R distribution and pathologies of chronic pelvic pain.

**Trial Registration:** The clinical trial registration date is June 2, 2018, and the registration number of the study is NCT03195270 (https://clinicaltrials.gov/ct2/show/NCT03556137).

## Introduction

Chronic pelvic pain is a common, debilitating medical condition affecting approximately 15% of women in westernized countries with rates ranging from 6 to 27% worldwide (1, 2). Chronic pelvic pain can be defined as persistent, non-cyclical pain lasting 6 months or longer. Multiple etiologies can contribute to pelvic pain, including gynecologic, gastrointestinal, musculoskeletal, urologic, neurologic, and psychosocial conditions. Unfortunately, studies have shown that up to half of the patients with chronic pelvic pain lack a clear diagnosis (3). While there have been recent improvements in magnetic resonance imaging and ultrasound diagnosis of endometriosis (4), adenomyosis (5, 6) and uterine fibroid pain (6), accurate diagnosis of one's pelvic pain remains a challenge. As a result, some cases of chronic pelvic pain lead to unnecessary and unhelpful procedures, suboptimal outcomes, and increased healthcare costs (2, 7). Additionally, half of the patients have more than one potential cause of chronic pelvic pain (8), which makes targeting the pain generator(s) challenging for treatment.

The most common gynecologic causes of chronic pelvic pain in women include endometriosis, prior pelvic inflammatory disease, ovarian cysts, adhesions, adenomyosis, and leiomyoma. Other etiologies include urologic, gastrointestinal, musculoskeletal, and neurologic conditions (1). Comprehensive algorithms for the diagnosis and treatment of chronic pelvic pain have been introduced by the European Association of Urology (9). If the diagnosis remains unclear after the physical examination and basic laboratory testing, patients with severe pain are suggested to have a laparoscopy. With laparoscopy, endometriosis and adhesions are the most commonly found diagnoses affecting 33 and 34%, respectively (1, 10). However, a lack of gross pathology on visual inspection is reported in up to 35–40% of those undergoing laparoscopy and the complex and multidisciplinary nature of chronic pelvic pain provides a challenge both diagnostically and for treatment. Additionally, laparoscopy is invasive and can lead to certain morbidities, and, therefore, a non-invasive imaging method to detect these structural and molecular abnormalities without the need for a surgical procedure is greatly desired.

Magnetic resonance imaging (MRI) is currently the non-invasive imaging modality of choice for many of the causes of pelvic pain, particularly for those of musculoskeletal and neurologic origin, and, as such, MRI is increasingly being used for gynecological evaluation. However, MRI is generally tailored toward a certain diagnosis or pathology, and the most common source of pelvic pain in women, endometriosis and adhesions, can be still challenging to identify on MRI. Prior studies have shown that MRI has high specificity but low sensitivity for detecting these pathologies, and up to 46% of patients with chronic pelvic pain that have no findings on MRI are found to have a gynecological structural cause on laparoscopy (11) or have multiple reasons of chronic pelvic pain that span different specialties and disciplines (8). Currently, no available clinical imaging or laboratory study is ideal given the vast possible etiologies of pelvic pain.

Sigma-1 receptors (S1Rs) are a unique class of intercellular chaperone proteins widely distributed throughout the whole body (12, 13). Increasing evidence suggests that S1Rs play a critical role in modulating ion channels and other neurotransmitter systems associated with pain and inflammation (14, 15). For example, elevated S1R expression was observed in response to inflammatory pain (16) while decreased pain-signaling activities and behavioral pain responses occurred with S1R antagonism (17, 18). [18F]FTC-146 (also known as S1R radioligand), is a highly specific radioligand that targets the S1R and enables non-invasive quantification of its expression through positron emission tomography (PET) (12). Therefore, S1R PET/MRI may facilitate the localization of increased activities of ion channels for pain-signaling, which can be exploited to identify associated pain generators. Our group has conducted a few preliminary studies to evaluate the feasibility of this S1R PET/MRI for the diagnosis of different pain conditions and demonstrated encouraging results in the detection of nerve injury (19), synovial lipoma of the knee (20), and a variety of potential musculoskeletal pain generators in complex regional pain syndrome (21) and radiating low back pain (22). The goal of our study is to conduct the initial evaluation of S1R PET/MRI for the diagnosis of chronic pelvic pain by comparing the S1R radioligand uptake in pelvic organs between chronic pelvic pain patients and asymptomatic volunteers.

## Materials and Methods

This prospective observational study was approved by the Stanford University Institutional Review Board, and all subjects signed a written informed consent form prior to imaging experiments. All data were collected in compliance with the Health Insurance Portability and Accountability Act. The clinical trial registration date is June 2, 2018, and the registration number of the study is NCT03195270 (https://clinicaltrials.gov/ct2/show/NCT03556137).

### Human Subjects

Five patients with chronic pelvic pain (age range 25–69) under the care of our OB/GYN surgeon (DL) and five healthy female controls (age range 28–49) were recruited. All patient subjects had had pelvic pain in the past 6 months at the time of referral to our study. To qualify for the study, patients had to report an average pain level of 4 or higher on a 0–10 visual analog scale (VAS) (23) on the day of the scan and in the past week. Exclusion criteria included diabetes, pregnancy or breastfeeding, severe comorbid conditions, severe claustrophobia, presence of MRI-incompatible materials/devices, diagnosed psychiatric disorder that would impede participation in the study, and inability to read or complete questionnaires in English. The PET/MRI scan was scheduled to avoid the menstruation period of the subject. At the time of the study, all patients completed surveys regarding the location and characteristics of their pain.

### S1R PET/MRI Process

Both patient and control participants fasted for 4 h before the PET/MRI scan and were injected with 10 mCi of [18F]FTC-146 via the antecubital vein in a bolus injection. 30 min after the injection, the simultaneous PET/MRI scan that covers the whole body in 8–10 bed positions was acquired using a hybrid GE SIGNA PET/MRI scanner (time-of-flight PET and 3T MRI; GE Healthcare, Waukesha, WI, U.S.A.). A combination of a head/neck coil, two anterior body array coils, and a spine coil was used for the signal reception. The following MRI sequences were performed for each bed position: 3D axial fast spoiled gradient-recalled echo with 2-point Dixon for fat-water separation (LAVA-FLEX) and 2D axial T2-weighted fast-spin-echo with triple-echo Dixon for fat-water separation (T2-FLEX). In the bed position for imaging the pelvis, we additionally adopted the following MRI sequences from the standard non-contrast pelvic MRI protocol in our clinical sites: 2D axial T1-weighted fast spin-echo sequence (T1-FSE), 2D axial T2-weighted fast spin-echo sequence with fat saturation (T2-FS-FSE), 2D sagittal T2-weighted PROPELLER sequence (T2-PROPELLER) (24), and 3D axial fat-saturated gradient-recalled echo sequence (FS-GRE). Detailed sequence parameters are described in Supplementary Table 1. Before and after the PET/MRI scan, we measured the blood pressure, heart rate, and oxygen saturation level of the subjects to find if there was any apparent side effect. We communicated with patients by email or text message for the following few days to identify whether there was any new development of adverse events, such as discomfort or pain, and how long they lasted if there was any.

### Image Analysis

Images were first reviewed independently by two radiologists (AF and SB) with at least 5 years of experience in reading pelvic MRIs, including looking for adhesions and endometriosis, to identify S1R-PET/MR abnormalities in comparison to healthy control subjects. The diagnostic pelvic MR images were first reviewed to identify pathology that could be contributing to the patient's pelvic pain symptoms. The PET images in the unit of standardized uptake value (SUV) were subsequently reviewed to look for marked abnormal uptake in comparison to the imaging pattern of the controls. The co-registered PET and MRI images were then reviewed using the patient surveys regarding the location and laterality of pain at the time of imaging. After the first review, radiologists consulted each other about PET/MRI findings, and they became congruent in the end.

The pelvic region was segmented into 5 organ types (uterus, vagina, pelvic bowel, pelvic muscle, liver) and the maximum SUV (SUV_max_) of [18F]FTC-146 was measured for each segmented region of individual control subjects. For each region, the mean and standard deviation of SUV_max_ across the five controls was calculated to evaluate the SUV_max_ of identified local hotspots of [18F]FTC-146 uptake from patients. The z-score of SUV_max_ for the identified hotspot was computed as $z=\;\frac{s-u}{\sigma }$, where z is the z-score, s is the SUV_max_ of the hotspot, and u and δ are the mean and standard deviation of SUV_max_ from the associated control organs among the 5 segmented types above. Image segmentation and SUV measurement were performed manually by the radiologists using Horos (https://horosproject.org/). The image findings were later matched to the follow-up treatment outcomes for validation, if available at the time of the image analysis.

## Results

### S1R Radioligand Uptake in Asymptomatic Controls

The mean ± the standard deviation of SUV_max_ was 3.9 ± 1.4 for the uterus, 3.2 ± 1.4 for the vagina, 2.3 ± 0.5 for the pelvic small bowel, 1.4 ± 0.4 for the pelvic muscle, and 2.4 ± 1.2 for the liver.

### S1R-PET/MRI Abnormalities

**Figure 1:**
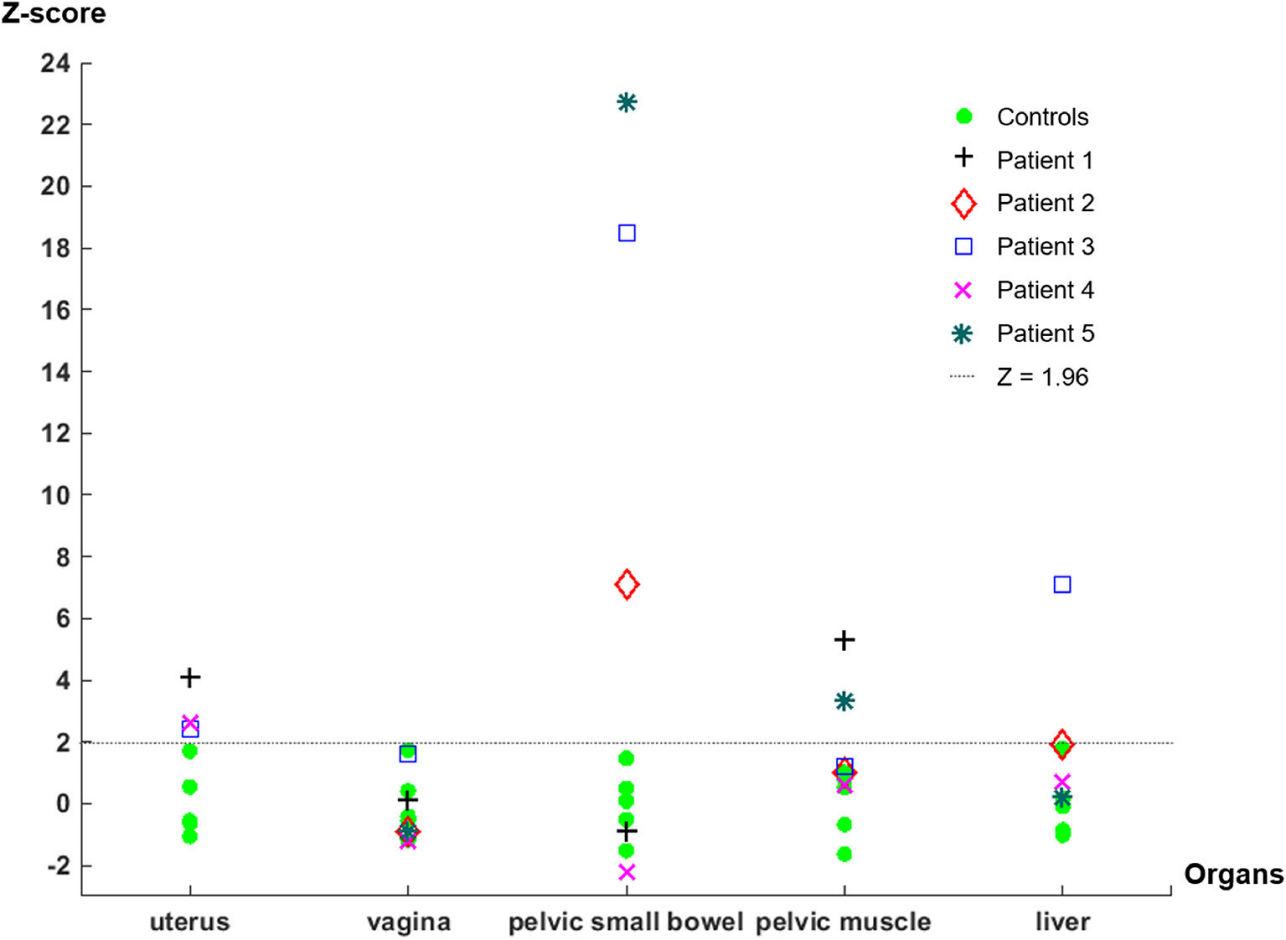
*Z*-scores derived from the SUV_max_ of each organ of control and patient subjects. Note that the uterus measurements of Patient 2 and Patient 5 are not available because the PET/MRI study was performed after they received hysterectomy. The liver uptake measurement of Patient 1 was not available because the liver was out of the FOV when the pelvic PET/MRI scan was acquired.

In all five patient participants, abnormalities with increased uptake of our S1R radioligand were identified during the image review as summarized in Table 1. However, only 2 of five patients presented abnormalities on MRI that could be related to the patient's pelvic pain symptom (Table 1). The z-scores derived from SUV_max_ of the identified abnormalities with increased uptake were significantly large (>1.96) except for those of increased vaginal uptake from Patient 3 and liver uptake from Patient 2. The z-scores of SUV_max_ from all patient and control subjects are plotted in Figure 1. Increased uptake in the uterus and pelvic bowel was the most common abnormalities on S1R PET (3 out of 5 patients). Abnormalities with increased uptake in the pelvic muscle, vagina, or liver were identified in one patient, respectively. On MRI, all findings were associated with the uterus (uterine fibroid, punctate foci of the adnexa), and no other abnormalities were detected.

**Table 1:** Abnormal S1R PET/MRI findings potentially associated with the patient's pelvic pain symptom.

**Patients**	**SUV_**max**_/z-score of abnormalities on PET**	**MRI abnormalities**
Pt. 1	9.5/4.1 (uterus) 3.6/5.3 (pelvic muscle)	Uterine fibroid
Pt. 2	5.8/7.1 (pelvic bowel) 4.6/1.9 (liver)	None
Pt. 3	7.2/2.4 (uterus) 11.5/18.5 (pelvic bowel) 5.5/1.6 (vagina)	Punctae foci in the adnexa
Pt. 4	7.4/2.6 (uterus)	None
Pt. 5	13.6/22.7 (pelvic bowel)	None

*On S1R PET, all five patients showed lesions with increased uptake of our S1R tracer in the symptomatic area, while, on MRI, abnormalities were found only in two patients*.

### S1R PET Image Examples

**Figure 2:**
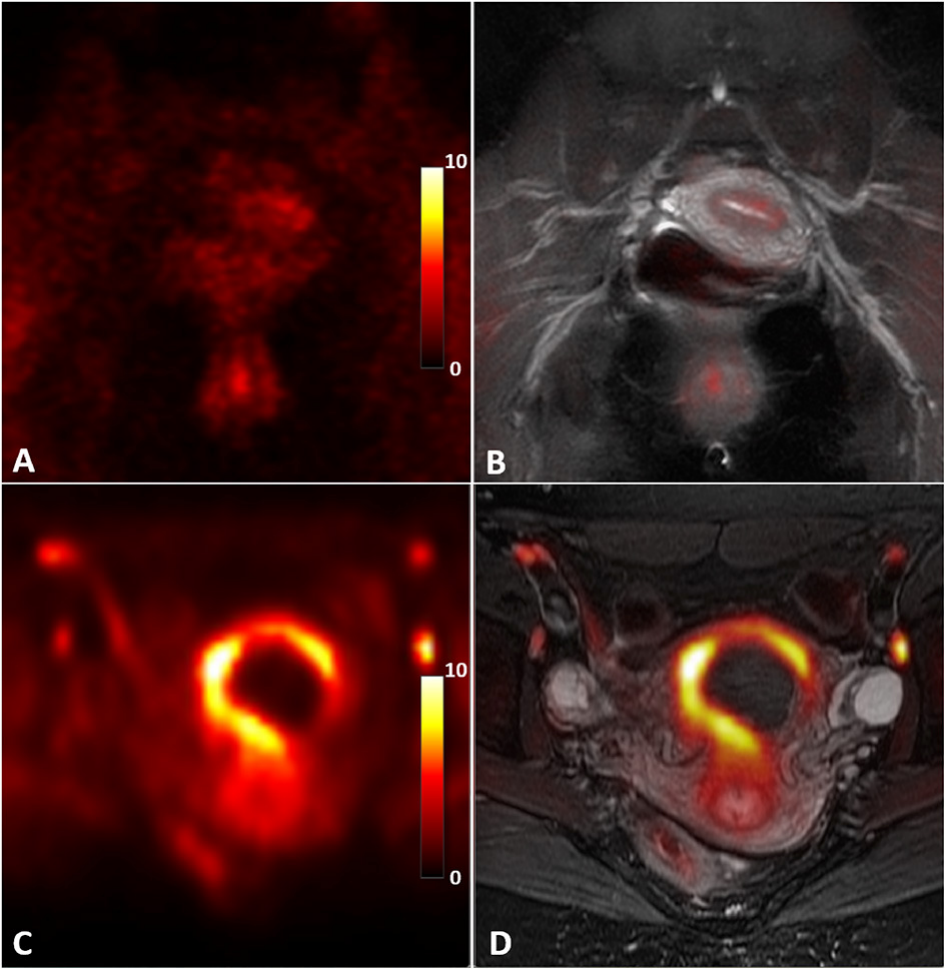
Abnormal S1R tracer uptake of the uterus in Patient 1 compared to an asymptomatic control. S1R PET **(A)** and PET/MR co-registered **(B)** images in an asymptomatic control present diffuse, low uptake compared to the S1R PET **(C)** and PET/MR co-registered **(D)** images of the patient. Note that the fibroid does not take up the radioligand. The PET images were plotted in the unit of standardized uptake value (SUV).

**Figure 3:**
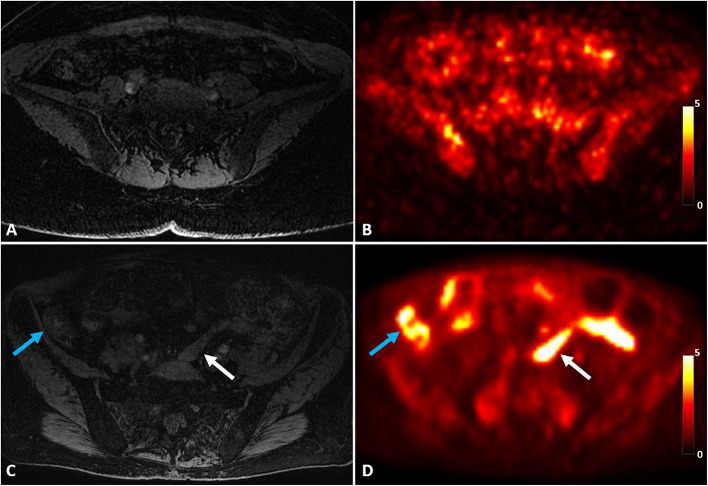
A representative control case of MRI **(A)** and S1R PET **(B)** of a healthy control in comparison with abnormal pelvic bowel uptake of Patient 2 on MRI **(C)** and S1R PET **(D)**. Increased S1R tracer uptake was identified throughout the bowel, including both within the bowel (white arrow) and around the bowel (blue arrow). The PET images were plotted in the unit of SUV.

Figure 2 shows an example of a uterus abnormality on S1R PET from Patient 1 in comparison with a representative case of healthy controls. Intense tracer uptake occurred throughout the uterus (SUV_max_ of 9.5) around the fibroid, which did not take up the tracer. The fibroid had been also shown to not enhance on previously performed clinical pelvic MRI with contrast. Figure 3 introduces an example of pelvic bowel abnormalities from Patient 2. The patient showed intense and diffuse radioligand uptake within and around the bowel with an SUV_max_ of 5.8. She previously underwent a laparoscopic hysterectomy and bilateral salpingo-oophorectomy for presumed endometriosis, however, her pathology only showed adenomyosis and India ink. Our imaging findings are hypothesized to potentially reflect underlying adhesive disease or an underlying bowel pathology. Supplementary Figure 1 presents the case of Patient 3, who was suspected to have vaginismus. Intense uptake within the vagina (SUV_max_ of 5.5) was observed, as compared to the healthy controls. Note that this patient also presented increased uptake in the uterus and pelvic bowel, fitting the suspected multifactorial causes of the symptom including endometriosis and adhesions.

### Post-imaging Follow-Up

No adverse event was identified during the post-imaging monitoring period. Patient 1 underwent a laparoscopic hysterectomy with pathology showing an infarcted fibroid and endometriosis in the bilateral pelvic sidewalls, left uterosacral ligament, and uterine serosa. These features would correlate with stage 2 endometriosis. A hysterectomy successfully resolved her pelvic pain, reducing the pain intensity from the VAS score of 8/10 before the surgery to the VAS score of 0/10 at the post-operative exam. Patient 3 ultimately underwent a diagnostic laparoscopy, cystoscopy, and vaginal trigger point injection. She was found to have one lesion of endometriosis in the left pelvic sidewall, which was excised and confirmed to be endometriosis on pathology. The finding was consistent with stage 1 endometriosis. Fortunately, her pain did improve post-operatively.

## Discussion

In this work, we presented the preliminary results of our S1R PET/MRI study on chronic pelvic pain patients. To our knowledge, this is the first study to demonstrate the feasibility of using S1R PET/MRI to identify potential pelvic pain generators. S1R has been previously reported to be upregulated on tissues under painful conditions to activate ion channels for pain-signaling. The purpose of our study is to investigate if our S1R PET/MRI may find differences between chronic pelvic pain patients and healthy controls, which might be used to locate pain generators. In our preliminary results, S1R PET images showed abnormalities that were likely associated with the pain symptom in all 5 patients while MRI showed potential abnormalities in only 2 of the 5 patients. Abnormally increased signal on S1R PET images was observed in different organs including the uterus, pelvic muscle, pelvic bowel, vagina, and liver compared to those of healthy controls. Our findings demonstrate that the proposed S1R PET/MRI approach can be used to examine the correlation of S1R and pelvic pain in a variety of pelvic organs.

MRI has been widely used when pelvic ultrasonography findings are unclear or when the spread of deep pelvic endometriosis should be assessed for presurgical planning (25, 26). It has been also useful for the diagnosis of inflammatory disease, pelvic congestion syndrome, and peripheral nerve compression (25). However, diagnostic laparoscopy was still deemed the gold standard for diagnosing endometriosis. A recent large-scale study compared the accuracy of MRI to laparoscopy in diagnosing structural causes of chronic pelvic pain, including deep pelvic endometriosis, superficial peritoneal endometriosis, endometriomas, adhesions, and ovarian cysts (11). MRI was found to be specific, particularly in detecting endometriomas and ovarian cysts, but MRI failed to detect pathology in up to 46% of patients that had significant findings at laparoscopy.

The pelvic MRI protocol in our study is composed to closely match the standard non-contrast pelvic MRI protocol adopted in the clinical sites of our institution. Though the sample size is limited, our result suggests that S1R PET may help revealing abnormalities that were previously undetected or very difficult to detect with MRI. Therefore, the proposed joint imaging approach of S1R PET and MRI may provide a more comprehensive and inclusive evaluation given the vast possible etiologies for pelvic pain. One of the most striking findings from our current work is the intense amount of uptake around the bowel in women who previously had extensive lysis of adhesions (Patient 2) and/or stage 4 endometriosis (Patient 5). Both of these entities are typically challenging to identify on MR imaging alone, and the adhesive disease tends to be a diagnosis of exclusion. Prior population studies have shown that between 50 and 61% of all women with pelvic pain lack a clear diagnosis (7, 27), and endometriosis and adhesions are the most common pathology documented on diagnostic laparoscopy for chronic pelvic pain (10).

There are a several limitations and future directions for this study. This is a small sample size with a variety of potential diagnoses, which limited the adoption of more rigorous statistical tests for the measurements and inter/intra-observer variabilities. The study of a larger control and patient cohort will be followed to conduct a randomized controlled trial to mitigate any potential bias in our case-control study format. Recruiting larger numbers of patients with known diagnoses would also help to validate the findings, and one goal is to recruit several patients with known diagnoses, such as endometriosis, adenomyosis, and fibroids, to see what the uptake patterns are for these diagnoses. The comparison with contrast-enhanced MRI scans (28, 29) will be needed to further evaluate the detection capability of the proposed S1R PET/MRI scan. The asymptomatic control sample size is also small, and it is currently unknown how the menstrual cycle would potentially affect the uptake of the S1R radioligand. Therefore, accurate matching of the menstrual cycles between subjects at the time of the imaging study needs to be implemented to reduce the influence from unrelated sources. Consistent pathologic confirmation of findings in this group of patients will be needed for the validation of identified abnormalities. For example, obtaining pathologic tissue samples during surgery and staining the tissues for SIR upregulation will be conducted in our future study to further assess the S1R binding efficiency of our radiotracer and the correlation of S1R expression with pelvic pain. Pelvic pain can be often referred as shown in the case of visceral pain (30), and thus the comparison of pre- and post-treatment imaging findings with the change of pain symptoms may help address the possible disconnection between the location of the lesion and pain symptoms. The scan participants should be monitored for a long term to detect any potential side effect from our radiotracer and establish its safety.

In conclusion, our early results demonstrate the potential use of S1R PET/MR in diagnosing pain generators in chronic pelvic pain. Given the many possible etiologies of chronic pelvic pain and the fact that many patients have more than one cause of their pain, this novel approach could help locate/distinguish specific pain generators and guide treatment based on molecular and structural findings. This molecular imaging of the sigma-1 receptor may also help identify conditions that are currently challenging to diagnose with the standard of care structural imaging, such as adhesive disease or distant endometrial implants, and guide/evaluate treatment, which would greatly benefit these patients.

## Data Availability Statement

The original contributions presented in the study are included in the article/Supplementary Material, further inquiries can be directed to the corresponding authors.

## Ethics Statement

The studies involving human participants were reviewed and approved by the Institutional Review Board of Stanford University. The patients/participants provided their written informed consent to participate in this study.

## Author Contributions

DL and SB: conceptualization, study design, and supervision. DY, PC, BS, JC, and CM: data acquisitions. AF, DY, and SB: drafting of the manuscript. SB and CRM: funding acquisition. All authors: formal analysis, investigation, and reviewing and editing.

## Funding

General Electric Healthcare provided research funding to DY, PC, and SB. This work was also supported by the University of Florida Clinical and Translational Science Institute, which was supported in part by the NIH National Center for Advancing Translational Sciences under award number UL1TR001427 (CRM).

## Conflict of Interest

DY, PC, and SB received research support from General Electric Healthcare. The remaining authors declare that the research was conducted in the absence of any commercial or financial relationships that could be construed as a potential conflict of interest.

## Publisher's Note

All claims expressed in this article are solely those of the authors and do not necessarily represent those of their affiliated organizations, or those of the publisher, the editors and the reviewers. Any product that may be evaluated in this article, or claim that may be made by its manufacturer, is not guaranteed or endorsed by the publisher.
