# Supplementary material for: Sigma-1 Receptor Changes Observed in Chronic Pelvic Pain Patients: A Pilot PET/MRI Study
Source: Front Pain Res (Lausanne). 2021 Oct 20;2:711748. doi: 10.3389/fpain.2021.711748 (PMC8915714; doi:10.3389/fpain.2021.711748)
Supplement: Supplementary file 1 [file Data_Sheet_1.docx]

Supplementary Material

|  | LAVA-FLEX | T2-FLEX | T1-FSE | T2-FS-FSE | T2-PROPELLER | FS-GRE |
| --- | --- | --- | --- | --- | --- | --- |
| TE | 1.2ms | 68ms | 9.7ms | 85ms | 104.1 | 4ms |
| TR | 5.0ms | 4s | 780ms | 6s | 8.6s | 8.8ms |
| FOV | 42x33.6cm | 38x30.4 | 26x52cm (no phase wrap) | 26x52cm (no phase wrap) | 30x45cm (no phase wrap) | 26x46.8cm |
| Frequency direction | R/L | R/L | A/P | A/P | A/P | A/P |
| Matrix size | 356x284 | 288x230 | 384x448 | 416x448 | 320x480 | 352x634 |
| Slice thickness | 2mm | 4mm | 4mm | 4mm | 5mm | 1.5mm |
| Flip angle | 15º | 160 º (refocusing RF pulse) | 111 º (refocusing RF pulse) | 111º (refocusing RF pulse) | 111 º (refocusing RF pulse) | 15º |
| Readout bandwidth | ±142.86kHz | ±166.67kHz | ±31.25kHz | ±31.25kHz | ±50kHz | ±31.25kHz |
| Number of excitation | 1 | 1 | 2 | 2 | 3 | 1 |
| Echo train length | N/A | 16 | 2 | 16 | 28 | N/A |
| Parallel imaging | 2x2 | 2x1 | 2x1 | 2x1 | 2x1 | 2x2 |

**Supplementary Table 1.** MRI pulse sequence parameters.


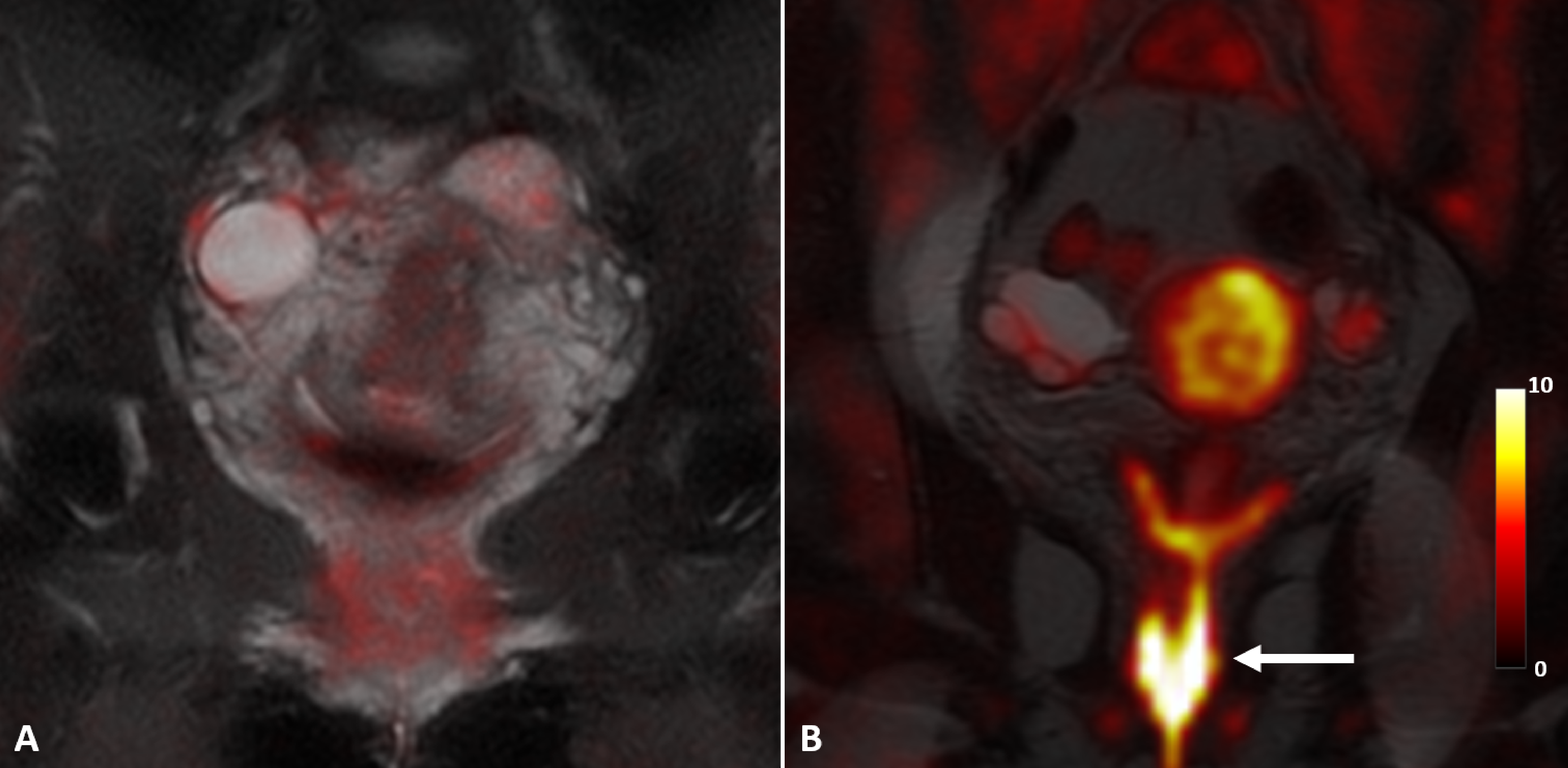


**Supplementary Figure 1.** A representative example of co-registered S1R PET/MR from a healthy control (A) and increased uptake of S1R tracer (white arrow) in the vagina of Patient 3 (B). The differential diagnosis of the patient included vaginismus, which supports the association of the increased uptake of our S1R tracer with the pain symptom. The PET images were plotted in the unit of SUV.
